# Nosocomial Valve Endocarditis after Crimean-Congo Hemorrhagic Fever

**DOI:** 10.1590/0037-8682-0691-2021

**Published:** 2022-06-06

**Authors:** Emine Parlak, Oğuzhan Birdal, Münacettin Ceviz

**Affiliations:** 1 Atatürk University, Faculty of Medicine, Department of Infectious Diseases and Clinical Microbiology, Erzurum, Turkey.; 2 Atatürk University, Faculty of Medicine, Department of Cardiology, Erzurum, Turkey.; 3 Atatürk University, Faculty of Medicine, Department of Cardiovascular Surgery, Erzurum, Turkey.

**FIGURE 2:**
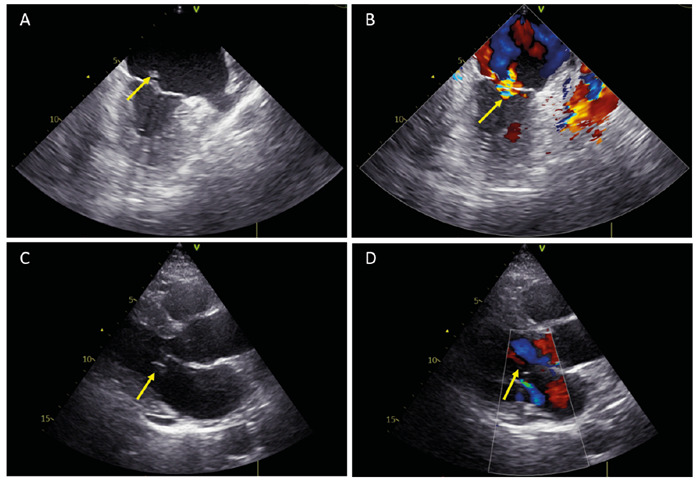
**(A)** Atransesophageal echocardiography image consistent with vegetation on the anterior mitral valve (1.2 x 0.4 cm). **(B)** A transesophageal echocardiography image consistent with mild mitral regurgitation. **(C)** A transthoracic echocardiography image consistent with vegetation on the anterior mitral valve (1.2 x 0.4 cm). **(D)** A transthoracic echocardiography consistent with mild mitral regurgitation.

A 59-year-old retired male presented with fever, lack of appetite, malaise, and general body pain 3 days after visiting a village in Oltu, Turkey. The patient was lucid, oriented, and cooperative. No findings other than hepatosplenomegaly were observed. No ticks were observed on his body, and he had no history of tick removal. The patient was negative for coronavirus disease performed by polymerase chain reaction at an external center. Crimean Congo hemorrhagic fever (CCHF) antibodies and immunoglobulin M (IgM) and IgG enzyme linked-immunosorbent assays were positive (Figure 1). No murmur was present during auscultation. Cultures were collected, and the patient was started on ampicillin, sulbactam, and daptomycin. A blurred vision was observed in the right eye. Transthoracic and transesophageal echocardiography were performed, and vegetation was detected on the anterior surface of the mitral valve (Figure 2). Growth of methicillin-susceptible *Staphylococcus aureus* was observed in the blood culture. Treatment with cefazolin was continued. The patient underwent surgery in the second month due to embolic attacks. He was then discharged in healthy condition. 

**FIGURE 1:** Laboratory values. **ALT:** alanine aminotransferase; **AST:** aspartate aminotransferase; **LDH:** lactate dehydrogenase; **CK:** creatine phosphokinase; **PT:** prothrombin time; **INR:** international normalized ratio; **CRP:** C-reactive protein

	Day 1	Day 2	Day 7	Day 9	Day 13
Hemoglobin g/dL	17.4	17	14.1	12.9	11.8
Leukocyte (/mm^3^)	2,070	1,440	10,510	8,950	7,690
Lymphocyte	520	440	880	1,350	1,630
Platelets (/mm3)	25,000	20,000	98,000	168,000	373,000
AST (U/L)	859	1,263	141	86	39
ALT (U/L)	533	680	121	79	30
LDH (U/L)	11,80	1,590	565	454	345
CK	4,336	2,290	360	167	165
PT (s)	13.4		20.8		
İNR	1.02		1.62		
CRP (mg/L)	14.81		84.16	258	122.9
Sedimentation	2		20	75	48

CCHF is a zoonotic infectious disease transmitted by ticks and endemic in Erzurum-Turkey^1^. The release of pro-inflammatory cytokines is thought to result in endothelial damage^2^. Infective endocarditis (IE) is an infectious disease with various manifestations. Staphylococci are most commonly involved in the etiology, with increased health services and invasive procedures^3^. 

CCHF should be considered in endemic regions. In addition, the number of nosocomial IE has increased. Therefore, more caution should be considered in using vascular catheters and invasive procedures. 
